# Classifying dynamic transitions in high dimensional neural mass models: A random forest approach

**DOI:** 10.1371/journal.pcbi.1006009

**Published:** 2018-03-02

**Authors:** Lauric A. Ferrat, Marc Goodfellow, John R. Terry

**Affiliations:** 1 College of Engineering, Mathematics and Physical Sciences, University of Exeter, Exeter, UK; 2 Living Systems Institute, University of Exeter, Exeter, UK; 3 Centre for Biomedical Modelling and Analysis, University of Exeter, Exeter, UK; 4 EPSRC Centre for Predictive Modelling in Healthcare, University of Exeter, Exeter, UK; The University of Melbourne, AUSTRALIA

## Abstract

Neural mass models (NMMs) are increasingly used to uncover the large-scale mechanisms of brain rhythms in health and disease. The dynamics of these models is dependent upon the choice of parameters, and therefore it is crucial to be able to understand how dynamics change when parameters are varied. Despite being considered low dimensional in comparison to micro-scale, neuronal network models, with regards to understanding the relationship between parameters and dynamics, NMMs are still prohibitively high dimensional for classical approaches such as numerical continuation. Therefore, we need alternative methods to characterise dynamics of NMMs in high dimensional parameter spaces. Here, we introduce a statistical framework that enables the efficient exploration of the relationship between model parameters and selected features of the simulated, emergent model dynamics of NMMs. We combine the classical machine learning approaches of trees and random forests to enable studying the effect that varying multiple parameters has on the dynamics of a model. The method proceeds by using simulations to transform the mathematical model into a database. This database is then used to partition parameter space with respect to dynamic features of interest, using random forests. This allows us to rapidly explore dynamics in high dimensional parameter space, capture the approximate location of qualitative transitions in dynamics and assess the relative importance of all parameters in the model in all dimensions simultaneously. We apply this method to a commonly used NMM in the context of transitions to seizure dynamics. We find that the inhibitory sub-system is most crucial for the generation of seizure dynamics, confirm and expand previous findings regarding the ratio of excitation and inhibition, and demonstrate that previously overlooked parameters can have a significant impact on model dynamics. We advocate the use of this method in future to constrain high dimensional parameter spaces enabling more efficient, person-specific, model calibration.

## Author summary

Understanding the workings of the healthy brain and the disruptions that lead to disease remains a grand challenge for neuroscience. Given the complexity of the brain, mathematical models are becoming increasingly important to elucidate these fundamental mechanisms. However, as our fundamental understanding evolves, so models grow in complexity. If the model has only one or two parameters, formal analysis is possible, however understanding changes in system behaviour becomes increasingly difficult as the number of model parameters increases. In this article we introduce a method to overcome this challenge and use it to better elucidate the contribution of different mechanisms to the emergence of brain rhythms. Our method uses machine learning approaches to classify the dynamics of the model under different parameters and to calculate their variability. This allows us to determine which parameters are critically important for the emergence of specific dynamics. Applying this method to a classical model of epilepsy, we find new explanations for the generation of seizures. This method can readily be used in other application areas of computational biology.

## Introduction

**Fig 1:**
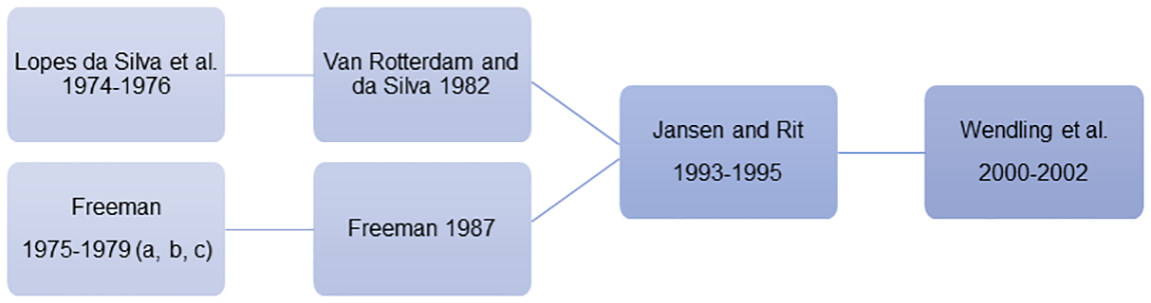
Historical development of the Wendling model. The history of neural mass models typically begins with the work of Lopes da Silva and Freeman in the 1970s, although strictly speaking it can be traced back to Beurle [27]. These classical works from 1970s were extended by van Rotterdam and Freeman during the 1980s, before the classical Jansen and Rit model of 1995. Wendling further extended this model in work at the turn of the millenium, extending the number of interneuron populations in the model. Interestingly, many of the parameter choices for the Wendling model in current use can be traced back to these early historic works.

Neural mass models (NMM) approximate the average behaviour of large populations of neurons and therefore provide an efficient way to simulate electrographic data in order to understand the mechanisms of brain (dys-) function. They have been used to understand a wide variety of physiological and pathophysiological activities of the brain, including the alpha rhythm [1, 2], sleep rhythms [3–5], brain resonance [6] or dynamics resulting from conditions such as epilepsy [7–11], schizophrenia [12] and dementia [13]. In particular, mechanisms underlying these conditions can be uncovered by inverting NMMs given dynamic data and studying the meaning of model parameters [14–17]. However, maintaining a sense of biological realism in NMMs results in a high dimensional parameter space. The presence of many parameters renders the estimation of parameters from data, or model inversion, a challenging task because it is difficult to systematically and exhaustively explore large hypervolumes in order to identify subvolumes that are plausible. In order to reduce dimensionality, subsets of parameters can be fixed based on *a priori* assumptions. Both the choice of initial values for parameters and the boundaries of the parameter space that are searched are often constrained [18]. Unfortunately, these constraints are often based on previously used values that have sometimes arisen arbitrarily in the literature. For example, the majority of parameters used in the study of [11] are taken directly from a previous study [19]. This study itself used previous parameter values [20, 21]. Ultimately these values were derived from studies made in the 70s [1, 22–26] (see Fig 1 for a summarised history of typically cited parameter values for the NMM). In these early derivations of NMM, parameters that could be experimentally determined were estimated but their uncertainties were not always measured [1].

Such parameters at the macroscopic level of NMM are often presumed to relate directly to properties of individual neurons but aggregated, for example, to mean values [28]. However, large variability has been shown to exist in parameters measured directly from neurons and even parameters that are considered to be quasi-certain in the modelling community, such as membrane time constants, have been shown to vary significantly in experiments [29]. Furthermore it remains unclear exactly how parameters of NMMs relate to microscopic properties of nervous tissue. Under standard values of NMM parameters, important insight has been gained regarding the generation of spontaneous or evoked electrographic recordings. For example, epileptiform rhythms have been shown to be induced by alterations to the excitatory/inhibitory balance in models [30]. However, fixing default values *a priori* in order to study the generation of particular dynamics does not allow to understand the behaviour of the system at unexplored, potentially plausible parameter values. Thus we cannot discover whether other regions of parameter space permit the same or different conclusions. When specifying prior distributions for model inversion (for example using the Kalman filter or Dynamic Causal Modelling frameworks [18, 31]) we usually, therefore, do not know to what extent any resulting inference is dependent upon the particular choice of priors or whether unexplored regions of parameter space could also provide reasonable solutions. High dimensionality of parameter space is a particular problem in such settings since inversion algorithms become computationally demanding. It is therefore often prohibitive to explore a large parameter space or conduct inference under alternative choices of priors. The same can also be said for the use of global non-deterministic searches, for example based on evolutionary algorithms [32].

Typically, the parameter space of NMMs contains nonlinear manifolds which delineate parameter sets that give rise to qualitatively the same dynamics. These manifolds can therefore be studied to understand the emergence of different dynamic regimes. Traditional approaches to mapping dynamics over changes in parameters include bifurcation analyses and simulation studies. Some models have been extensively studied via these methods [33, 34], which typically only examine two parameters simultaneously. Clearly, in high dimensional systems such as NMM, we expect that changing a third parameter could affect the distribution of dynamics obtained. As such, the Jansen model [19] have been studied comprehensively by simultaneously altering 3 parameters [30]. A potential downside to such analyses is that results can be cumbersome and difficult to summarise, thus moving beyond 3 parameters with these techniques would prohibit a succinct evaluation of the role of each of parameter. Another approach is to extend multiple bifurcation analyses in a single parameter across further dimensions, whilst classifying different bifurcations and their prevalences [35]. Although this is a valid approach to understanding some elements of the complexity over large dimensional parameter spaces, it does not give a comprehensive overview of the role that each parameter plays. Even if very enlightening about the role and codependence of a few parameters, bifurcation theory cannot be use to simultaneously approach all parameters. In high dimensions (e.g. D ≥ 3) these methods soon become computationally intractable and are not able to characterise the effect on dynamics of changing all parameters simultaneously. On the other hand, studying a restricted number of parameters is unsatisfactory.

It would therefore be highly beneficial to develop approaches to understand the repertoire of NMM dynamics over all parameters that cannot be sufficiently constrained. Such an approach would facilitate choosing appropriate priors and initial parameter settings in model inversion algorithms. It would also facilitate a deeper understanding of complex, high dimensional models. Approaches such as global sensitivity analyses (variance-based methods [36], screening [37] or generalised models [38]) have previously been used to identify the existence of relationships between dynamics and parameters. However, these methods do not allow to quantify the impact that changes in multiple (e.g. all) parameters have on dynamics, or to identify specific regions of parameter space in which changes in dynamics occur.

In this study we therefore introduce a new methodology for the characterisation of NMM dynamics simultaneously over all parameters. The NMM is simulated a large number of times with different combination of parameters. The simulations are then classified according to their dynamics using pre-specified features. The relationships between parameter space and dynamics are then studied using the classical machine learning method of decision trees and random forests [39]. The decision trees can map the parameter space and the random forest can be used to rapidly characterise the dynamics of the NMM under previously unexplored parameter combinations. Importantly, the resulting statistical model yields natural means by which to quantify the relative importance that each parameter plays in the generation of dynamic characteristics of interest, without restricting analyses to low dimensional subspaces. We use this method to demonstrate the significance of previously overlooked NMM parameters for both physiological and pathophysiological rhythms.

## Methods

### Overview

**Fig 2:**
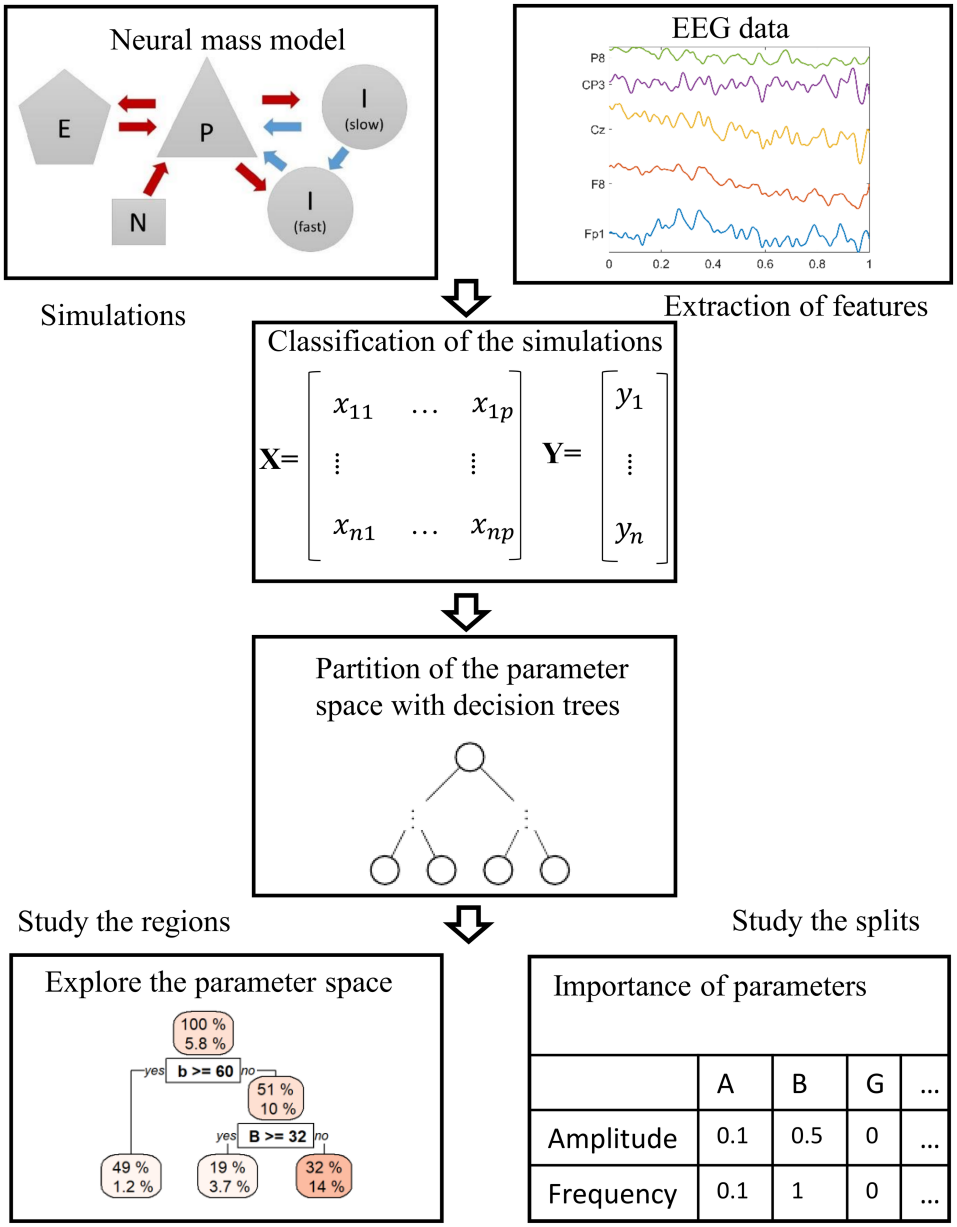
Schematic of the methodology. First, the dynamic features of interest are identified and characterised (this could be from a model or as in this case from data. The NMM is simulated a large number of times over its parameter space. Next, each simulation is given a classification according to its dynamic features. Then the simulations are partitioned using decision tree learning. The final partitions are used to characterise the parameter space of the NMM.

In this section we give a brief descriptive overview of the approach and provide further mathematical details of each component in subsequent sections. To do so, we transform the NMM into a statistical model, which is a function that maps parameters onto a quantification of these important features. This statistical model can then be analysed to understand the relationship between the NMM parameters and the dynamics (see Fig 2 for a general overview). The first step consists of choosing a NMM and defining a plausible parameter space, i.e. some constraints on the extreme values that each parameter can take. In this study we use a variation of the Jansen and Rit model introduced in the context of epilepsy [11]. This model, which we refer to as the Wendling model, has 11 parameters. The second step in the methodology consists of transforming the mathematical model into a database. To do this the NMM is simulated a large number of times using different parameters, which are chosen using a latin hypercube design. This is a space filling design which allows to efficiently explore the whole parameter space given a fixed number of simulations [40]. Each simulation is then classified in terms of some chosen characteristics. Here, we choose to focus on characteristics that are often used to define healthy and epileptiform rhythms, i.e. amplitude, frequency and number of peaks per period. The amplitude was defined as the maximum minus the minimum of the simulation. In cases for which the amplitude was greater than zero, i.e. the simulation was not constant, the frequency of the cycle and the number of peaks per period were calculated. The number of peaks can be used, for example, to characterise pathological dynamics. One of our aims is to characterise qualitative changes in model dynamics over the features above, since such an approach would enable us to find boundaries in parameter space over which dynamics change. We therefore seek to “classify” dynamics, rather than, for example, estimate quantitative features. Studying the database with classical statistics such as the joint distribution of the likelihood of seizure dynamics gives new insights into the model, but does not yield a comprehensive analysis.

The final step is to fit the data with a statistical model. Here, we choose to use a tree approach, which cuts the parameter space into rectangular regions of different sizes and is amenable to high dimensional analyses. These regions are created with the aim that each one contains similar dynamics and so trees approximate the parameter space in a simple and interpretable way. Of course, we do not expect that the parameter space can be completely mapped to a set of rectangular regions, each containing homogeneous dynamical features. Some regions therefore contain dynamics with different features and the proportion of space in the region filled by particular dynamics is useful information. For example, one can ask whether certain regions contain a high density of seizure dynamics or exclude regions with certain features from further analyses. The statistical model captures defined characteristics of the mathematical model and summarises them in an efficient way, therefore facilitating the estimation of sensitivity of the dynamics to variations in a particular parameter. Thus critical, or important parameters for a given dynamics can be found.

### Wendling model

The extension of the Jansen-Rit model [19] introduced by Wendling et al. [41] considered in this paper has classically been used to study transitions to seizure dynamics. It is a neurophysiological model, i.e. one that has been built to understand interactions in nervous tissue at the macro- or meso-scopic level. It has previously been shown to display a repertoire of important dynamics which occur at ictal and inter-ictal states, for example in temporal lobe epilepsy [11, 41]. The model is based on the assumption of the existence of four populations of neurons: pyramidal cells; excitatory interneurons; slow and fast inhibitory interneurons. The activity of each population is governed by the interactions between them. Each population is characterized by:

1. Its second order linear transfer function. This function transforms the average presynaptic pulse density of afferent action potentials of other populations of neurons (the input) into an average postsynaptic membrane potential (the output). This can be either excitatory, slow inhibitory or fast inhibitory with respective impulse responses *h*_*e*_(*t*), *h*_*i*_(*t*) or *h*_*g*_(*t*).
2. A sigmoid function $S\left(v\right)=\frac{2e_{0}}{1+\text{exp}\;\left(r\left(v_{0}-v\right)\right)}$ that relates the average postsynaptic potential of a given population to an average pulse density of action potentials outgoing from the population.

The total potential of the pyramidal cell population is given by the aggregated contributions of the three feedback loops of inter-neurons connected to it. This is the output of the model, in analogy with recordings of electroencephalography (EEG) [42]. These interactions can be summarise in the following set of ordinary differential equations:

$${\dot{z}}_{1}\left(t\right)=z_{6}\left(t\right)$$ (1)

$${\dot{z}}_{6}\left(t\right)=AaS\{z_{2}\left(t\right)-z_{3}\left(t\right)-z_{4}\left(t\right)\}-2az_{6}\left(t\right)-a^{2}z_{1}\left(t\right)$$ (2)

$${\dot{z}}_{2}\left(t\right)=z_{7}\left(t\right)$$ (3)

$${\dot{z}}_{7}\left(t\right)=Aa\left(p+C_{2}S\{C_{1}z_{1}\left(t\right)\}\right)-2az_{7}\left(t\right)-a^{2}z_{2}\left(t\right)$$ (4)

$${\dot{z}}_{3}\left(t\right)=z_{8}\left(t\right)$$ (5)

$${\dot{z}}_{8}\left(t\right)=BbC_{4}S\{C_{3}z_{1}\left(t\right)\}-2bz_{8}\left(t\right)-b^{2}z_{3}\left(t\right)$$ (6)

$${\dot{z}}_{4}\left(t\right)=z_{9}\left(t\right)$$ (7)

$${\dot{z}}_{9}\left(t\right)=GgC_{7}S\{C_{5}z_{1}\left(t\right)-z_{5}\left(t\right)\}-2gz_{9}\left(t\right)-g^{2}z_{4}\left(t\right)$$ (8)

$${\dot{z}}_{5}\left(t\right)=z_{10}\left(t\right)$$ (9)

$${\dot{z}}_{10}\left(t\right)=BbC_{6}S\{C_{3}z_{1}\left(t\right)\}-2bz_{10}\left(t\right)-b^{2}z_{5}\left(t\right)$$ (10)

### NMM parameters

The biological meaning of the NMM parameters is given in Table 1. As highlighted in the introduction the values of these parameters or their possible ranges are often based on previously used values that have sometimes arisen arbitrarily in the literature. As further experiments are conducted over time, it is possible to gain an improved insight into the range that NMM parameters could take. Examination of the experimental literature reveals that neuronal level mechanisms, which are often assumed to map to NMM parameters, can vary significantly from one species to another, as well as within species [29] (neuroelectro.org). Therefore the plausible range of NMM parameters can be large. The parameters *A*, *B*, *G*, *C* and *P* have traditionally been considered to be highly uncertain and dynamics have therefore been studied over substantial ranges of these parameters [11, 19, 43]. In contrast, the membrane time constants *a*, *b* and *g* have often been considered as relatively certain [11, 19]. However, experimental studies point towards the contrary. For example, there is a large uncertainty of dendritic time constants of the somatic response due to synaptic input for single neurons [44, 45]. Ranges for these values have been shown to be large, from 25 s^−1^ [46] to 140 s^−1^ [47] for pyramidal neurons. Similarly, the membrane time constant of inhibitory neurons (related to *b*) could also be considered uncertain, with values ranging from 6.5 s^−1^ to 110 s^−1^ [48]. We use these experimentally determined ranges for values of *a* and *b* in our study (see Table 2). It is more difficult to find a plausible range for *g*; values used can be traced back to 1993 [49], in which the authors indicated a large uncertainty. We therefore implement a large range for this parameter (350 to 650^−1^). *C* was previously fixed at 135 [19] based on interesting dynamics occurring near this value. Here, we chose to use the initial range of uncertainty in [19] from 0 to 1350. v_0_ was considered uncertain in previous studies and has also therefore been examined across a range of values, for example 2 to 6 mV [19]. Here, we extend the study from 2 to 10. e_0_ is often fixed at 2.5 s^−1^ but a range from 0.5 to 7.5 s^−1^ has been recorded [24], and therefore we use this range. Finally there is very little information about *r*, the value was found experimentally, but without information regarding uncertainty [23]. We therefore studied the range of this parameter from 0.3 to 0.8 mV. A summary of ranges of parameter values implemented in our study is given in Table 2.

**Table 1:** Description of parameters in the Wendling model.

Parameter	Interpretation
A	Average excitatory synaptic gain
B	Average slow inhibitory synaptic gain
G	Average fast inhibitory synaptic gain
a	Inverse mean time in the excitatory loop
b	Inverse mean time in the slow inhibitory loop
g	Inverse mean time in the fast inhibitory loop
P	Input to the system from the area of the cortex
C_1_	Connectivity pyramidal to excitatory
C_2_	Connectivity excitatory to pyramidal
C_3_	Connectivity pyramidal to slow inhibitory
C_4_	Connectivity slow inhibitory to pyramidal
C_5_	Connectivity pyramidal to fast inhibitory
C_6_	Connectivity slow inhibitory to fast inhibitory
C_7_	Connectivity fast inhibitory to pyramidal
v_0_	the postsynaptic potential for
	which a 50% firing rate is achieved
e_0_	1/2 maximum firing rate of the neural population
r	Steepness of the sigmoidal transformation

**Table 2:** The range of considered parameter space of the Wendling model. Details of the reference used to define the minimum and maximum value of each parameter is included. Chosen ranges were constrained either by experiments (e.g. *a* and *b*) or the widest range described in theoretical studies (e.g. *P* and *C*).

parameter	nominal value	min	max	Reference
A	5 mV	0	10	[19, 33]
B	22 mV	0	50	[19, 33]
G	20 mV	0	50	[19, 33]
P	90 spikes.s^−1^	0	2000	[43]
a	100 s^−1^	25	140	[46, 47]
b	50 s^−1^	6.5	110	[48, 50]
g	500 s^−1^	350	650	[11]
C	135	0	1350	[7, 19]
v_0_	6 mV	2	9	[19]
e_0_	2.5 s^−1^	0.5	7.5	[24]
r	0.56 mV^-1^	0.3	0.8	[23]

### Model simulations

The NMM was simulated 2,000,000 times varying 11 parameters *A*, *B*, *G*, *P*, *a*, *b*, *g*, *C*, v_0_, e_0_ and *r* using a latin hypercube design to explore the parameter space. The simulations were computed using ODE45 in MATLAB (Runge–Kutta method).

Each time, 20 seconds of EEG activity were simulated, the first 10 seconds were removed to eliminate transients. Simulations were performed in parallel over 4 CPUs each running at 3.5 GHz. It took approximately 4 days to simulate the whole data base (i.e. 2,000,000 simulations).

### Quantifying dynamic transitions in high dimensions

**Fig 3:**
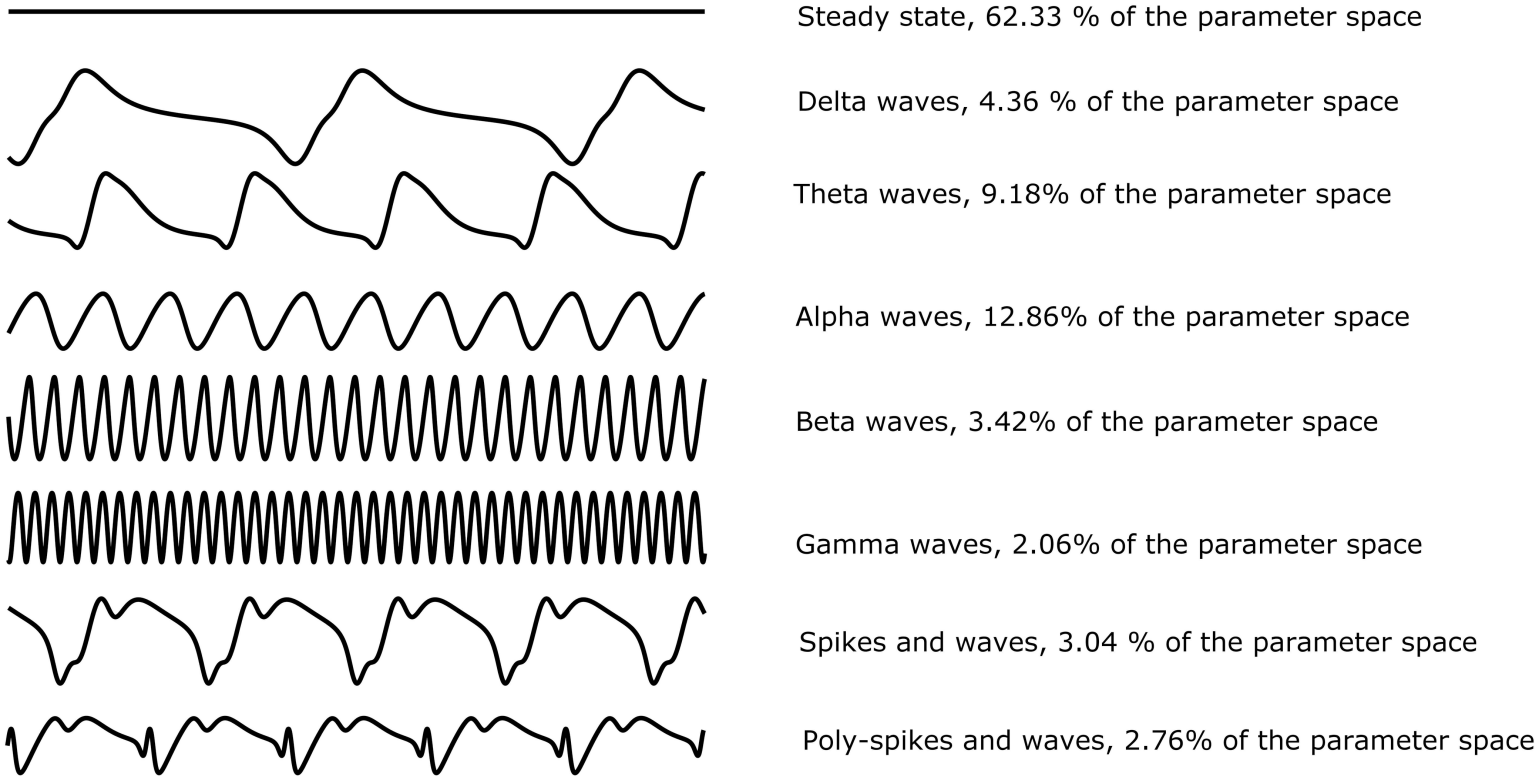
Common dynamic patterns observed in the Wendling model. Non-steady-state solutions are split into two categories: oscillations and (poly)spike-wave dynamics. Oscillations are cycles with one peak per period delineated by frequency into the five classical clinical bands: gamma (30-60Hz); beta (13-30Hz); alpha (8-12Hz); theta (4-8Hz); and delta (0-4Hz). (Poly)spike-wave dynamics are cycles with one or more spikes per period, riding on an oscillation of between 2 and 8 Hz with a mean of 4 Hz. For the sake of clarity amplitudes do not have uniform y-axis scales.

We are interested in understanding the relationship between parameters of the NMM and its dynamics. This understanding can be achieved through an explicit mapping between regions of parameter space and qualitatively different dynamics (e.g. steady states and oscillations). Previous studies have analysed the dynamics of NMMs by characterising features of simulations. Different properties of dynamics have been used for characterisation, such as the power spectrum [11], amplitude or variance [51, 52] and more nuanced features such as the number of spikes within a period of a specific rhythm [32, 33]. These studies have demonstrated that NMMs can recreate key types of epileptiform dynamics such as slow spike-wave rhythms and theta spikes, which are important rhythms for generalised and focal epilepsies, respectively. Based on these previous studies, we consider three key features of simulations that are relevant for delineating different types of dynamics within the NMM: *amplitude*, *frequency* and *number of peaks per cycle*. We use these features to classify regions of parameter space according to the nature of the emergent dynamics. For example, alpha activity corresponds to low-amplitude oscillations with a frequency of around 10Hz. Alternatively, seizure dynamics in this model correspond to low-frequency oscillations (2-8Hz to take into account focal and generalized seizure activity) with additional peaks that correspond to “spikes” or “poly-spikes” in EEG (c.f. Fig 3).

We formalise this idea by denoting *P*(*Y*|*X* ∈ *R*) as the probability of the dynamics *Y* given that the parameter set *X* belongs to region *R*, which is a hypercube subset of the full parameter space. For example we could have:

$$\begin{array}{cc} Y= & \{\begin{array}{ll} 0, & \text{if}\;\text{the}\;\text{dynamic}\;\text{state}\;\text{of}\;\text{interest}\;\text{is}\;\text{seizure}\;\text{dynamics};\\ 1 & \text{otherwise}. \end{array} \end{array}$$

and a region defined, for example, by

$$\begin{array}{c} X\in R=X\in ((A<5)\cap (15<B<30)). \end{array}$$

In this case *P*(*Y* = 1|*X* ∈ *R*) represents the likelihood of observing seizure dynamics when the parameter *A* is inferior to 5mV, *B* is between 15 and 30 mV and the other parameters are not constrained. The value of *P*(*Y* = 1|*X* ∈ *R*) is given by

$$\begin{array}{c} P\left(Y=1|X\in R\right))=\int_{x\in R}P\left(Y=1|x\right)\;dx \end{array}$$ (11)

Since the function mapping *X* onto *Y* is unknown, we take a sampling approach and use the database created by the simulations defined in the section Wendling model.

We can therefore estimate *P*(*Y* = 1|*X* ∈ *R*) for the given region *R* by

$$\begin{array}{c} \hat{P}\left(Y=1|X\in R\right)=\frac{1}{\left|\chi \right|}\sum_{\chi }^{}y. \end{array}$$ (12)

where *χ* = {*x*|*x* ∈ *R*} and |*χ*| denotes the cardinality of the set. *P*(*Y* = 1|*X* ∈ *R*) can be further used to determine which parameters are important to find certain dynamic regimes.

An obvious question that arises is how to choose *R*. A first approach consists of fixing the regions *R*_*i*∈[1:*m*]_ such that each region has the same size, i.e. the parameter space is cut into pre-defined regions.

Another approach consists of partitioning the parameter space by selecting *M* “optimal” regions, *R*_1_, …, *R*_*M*_. By optimal, we mean the number of regions *M* is as small as possible such that in each region the discrepancy of the event *Y* is low. In principle, this results in a more efficient mapping of the dynamics of the model onto its parameter space. Furthermore the boundaries between regions are useful, as they indicate which parameters have an important role in the emergence of dynamics of interest. Effectively they describe the transitions between different dynamic types that can correspond to bifurcations or other types of phase transition in the underlying dynamic model.

To define optimal regions, we use an approach called decision tree learning algorithms [53]. Here, the parameter space is partitioned recursively into rectangular disjoint subspaces. The size of each region is determined by ensuring that it consists, as far as possible, of only a single type of dynamics. Tree-based methods are a conceptually simple, yet very powerful tool to study highly nonlinear functions for the purpose of regression or classification. These methods are inherently non-parametric; no assumptions are made regarding the underlying distribution of parameter values. They can be trained quickly and also provide a vehicle to efficiently predict the output of new simulations. We focus on classification and regression tree (CART) algorithms [53]. These produce binary splits recursively from the root (the complete parameter space), to its leaves (the regions corresponding to dynamics of a single type).

### Building a tree

In general, finding the optimal partitioning of parameter space is a NP-complete problem [54]. Therefore, decision tree learning algorithms are based on heuristics whereby locally optimal decisions are made within each region of the tree. Whilst such an approach is not guaranteed to give the globally-optimal decision tree, CART methods have been shown to give good results in practice [53]. Here, we summarise the approach, which is described in detail in [39]. Formally we have a data set consisting of *n* points in $R^{p}$, *x*_*ij*_ where *i* ∈ [1: *n*] and *j* ∈ [1: *p*]. The set of outputs consists of the class of observed dynamics *Y*_*i*_ of each simulation. Suppose that we have a partition into *M* regions, *R*_1_, …, *R*_*M*_. For a given region *R*_*m*_ the splitting stage is chosen by finding an optimal split point in terms of the impurity criterion described Eq (14).

We seek the *j*^th^ split parameter and split point, *s*, such that the cost function:

$$\begin{array}{c} \underset{\left\{s,j\right\}}{\mathrm{argmin}}\;\;I_{R_{L}\left(m,j,s\right)}+I_{R_{R}(m,j,s)} \end{array}$$ (13)

is minimised. Here *R*_*L*_(*m*, *j*, *s*) = {*x*|*x* ∈ *R*_*m*_, *x*_.*j*_ ≤ *s*} and *R*_*R*_(*m*, *j*, *s*) = {*x*|*x* ∈ *R*_*m*_, *x*_.*j*_ > *s*} are respectively the potential left and right split of the region of interest. The measure $I_{R_{m}}$ of region impurity represents the quality of classification in a region. By this we mean how well a region of parameter space maps onto model dynamics of a single type. It is defined by the Gini index

$$\begin{array}{c} I_{R_{m}}=\frac{1}{N_{m}}\sum_{k=1}^{K}{\hat{p}}_{mk}\left(1-{\hat{p}}_{mk}\right) \end{array}$$ (14)

where

$$\begin{array}{c} {\hat{p}}_{mk}=\frac{1}{N_{m}}\sum_{x_{i.}\in R_{m}}I\left(y_{i}=k\right) \end{array}$$ (15)

is the proportion of class *k* observations in a given region *R*_*m*_. When *I*_*R*_ = 0 the region is pure, and there is only a single class of dynamics. By contrast a large Gini index indicates a region with large impurity, and thus contains parameters that map onto different types of dynamics in the model.

**Fig 4:**
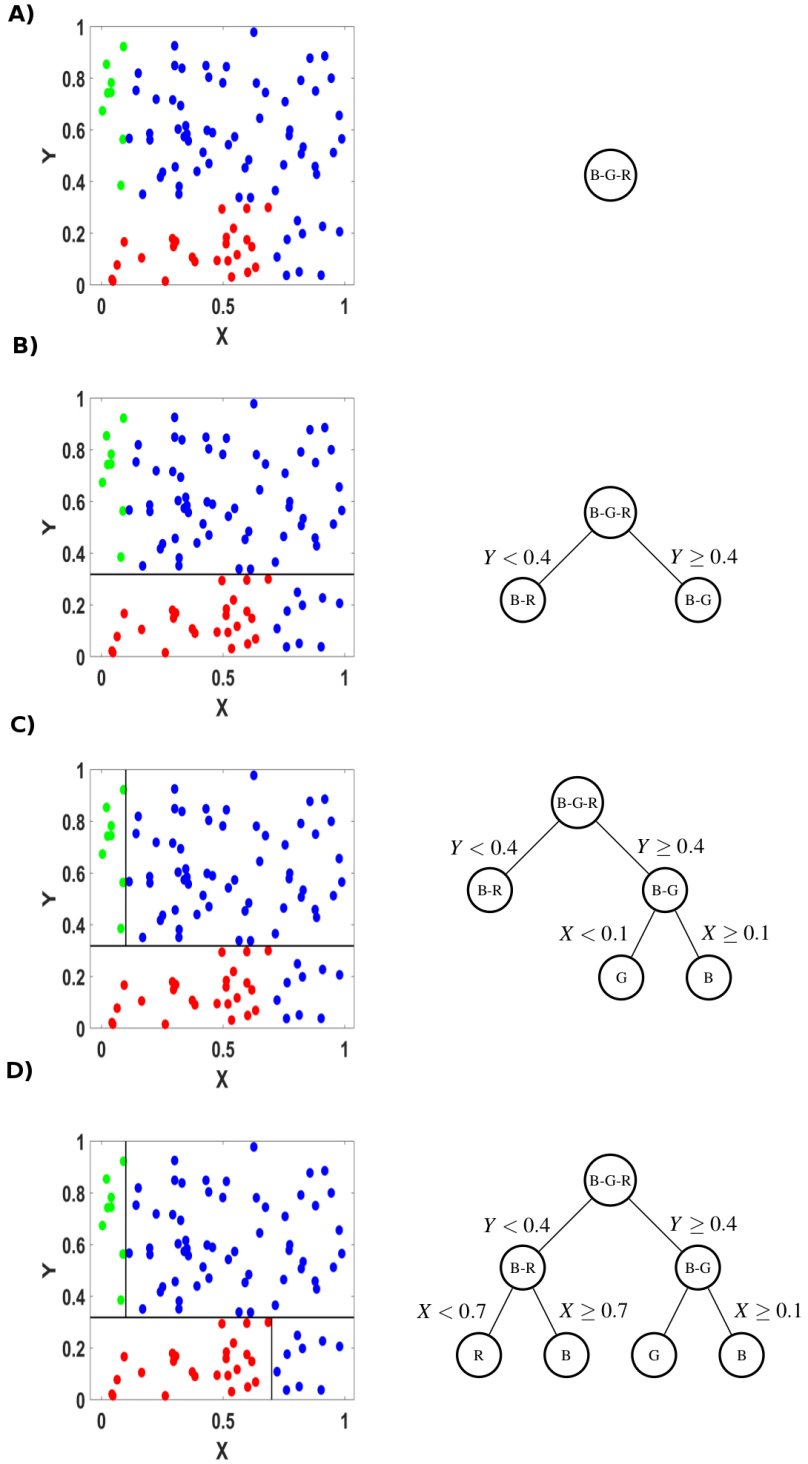
The different steps to construct a tree. In this example, the parameter space consists of two parameters *X* and *Y* ranging from 0 to 1. There are 3 classes of dynamics. A) This full parameter space is represented in the tree by its root. In this region all classes are represented. The impurity in this region is equal to 0.48. B) The split at *Y* = 0.4 drastically removes the impurity in its two sub regions which are now 0.13 and 0.14. Nevertheless the regions themselves are not pure. C) The region with the larger impurity is then targeted by the CART algorithm. The split at *X* = 0.1 totally removes the impurity in its two sub regions which are now equal to 0. There is only one type of observation per region in this part of the parameter space. D) The last region with impurity is again targeted by the CART algorithm. The split at *X* = 0.7 totally removes the impurity in its two sub regions. There is now only one type of dynamic feature within each region of the tree.

For each region, the determination of the split points can be done very quickly (*o*(*p* × *n*) operations) and hence by scanning through all of the inputs, determination of the best pair (*j*, *s*) is feasible in finite time. An example of a tree and its construction can be found in Fig 4. To estimate a new set of parameters *x*, the class with the largest frequency $k\left(m\right)=\underset{k}{\mathrm{argmax}}\;{\hat{p}}_{mk}$ is attributed to *x*. Furthermore $\hat{P}\left(y\in k\right)={\hat{p}}_{mk}$.

#### Random decision forests

Problems faced when focussing on a single tree include overfitting and the inability of the heuristic to find the optimal partition. To overcome these problems, the aggregation of a large number of trees is often used, and provides much greater insight. In a series of papers and technical reports, [55–57] demonstrated that substantial gains in classification and regression accuracy can be achieved by using ensembles of *B* trees, where each tree in the ensemble is grown in accordance with a random set of rules. This method is called random decision forests. This method remains one of the most accurate machine learning algorithms [58, 59].

In this study the use of multiple trees is equivalent to mapping the parameter space using different rules of segmentation. If a segmentation appears consistently, this implies it is important. The training algorithm for random forests applies the general technique of bootstrap aggregating [60], i.e. for each tree, a random sample with replacement of the training set is selected. Furthermore for each region, a random subset of parameters is selected and the split is optimised on the basis of the chosen parameters. For each bootstrap sample Z*^b^, {*b* = 1, 2, …, *B*}, we fit a tree according to a succession of random rules *r*, giving the tree t*^b^. Then the random forest *f* is given by:

$$\begin{array}{c} f=\frac{1}{B}\sum_{b=1}^{B}t^{*b} \end{array}.$$ (16)

The estimation of the probability for a set of parameters *x* to belong to class *k* is given by:

$$\begin{array}{c} \hat{P}\left(y\in k\right)=\frac{1}{B}\sum_{b=1}^{B}{\hat{P}}_{b}\left(y\in k\right). \end{array}$$ (17)

#### Determining the importance of a parameter

Knowing which parameters have a high impact on the dynamics of a model is crucial to improve our understanding of the system so that we may focus on these parameters.

The variable importance *VI*(*j*), *j* ∈ [1: *p*] (also called Gini Importance in [61]) quantifies how much the dynamics depend on the parameter value. We note that in the statistical literature, parameters are often termed “variables” since it is implicit that we would study the effect of changing their values. This is in contrast to the study of dynamical systems, in which parameters are considered to have fixed values relative to the evolution of “variables”, for which the differential equations are defined. Here, the variable importance is defined as the sum of all decreases in impurity in the tree due to the given parameter divided by the number of branch regions, *Nb*, i.e.

$$\begin{array}{c} VI\left(j\right)=\frac{1}{Nb}\sum_{R_{m}\in T}I_{R\left(m\right)}-I_{R_{L}\left(m\right)}+I_{R_{R}\left(m\right)} \end{array}$$ (18)

A parameter with a large *VI* indicates that a change in the values of the parameter is more likely to influence the dynamics than a parameter with small *VI*. The variable importance *VI*(*i*) of the parameter *i* is expressed in terms of a normalized quantity relative to the variable having the largest measure of importance.

$$\begin{array}{c} NVI\left(i\right)=\frac{VI(i)}{max(VI)} \end{array}$$ (19)

A parameter therefore has an important influence on the dynamics of interest in the model if its *NVI* is close to 1 and a small importance if it close to 0. These values are only indicative and small differences in *NVI* between two parameters would not necessarily indicate that a parameter is more important than another. Furthermore it quantifies *global* parameter importance; it is possible that in some parts of the parameter space a parameter described as important does not affect dynamics.

**Fig 5:**
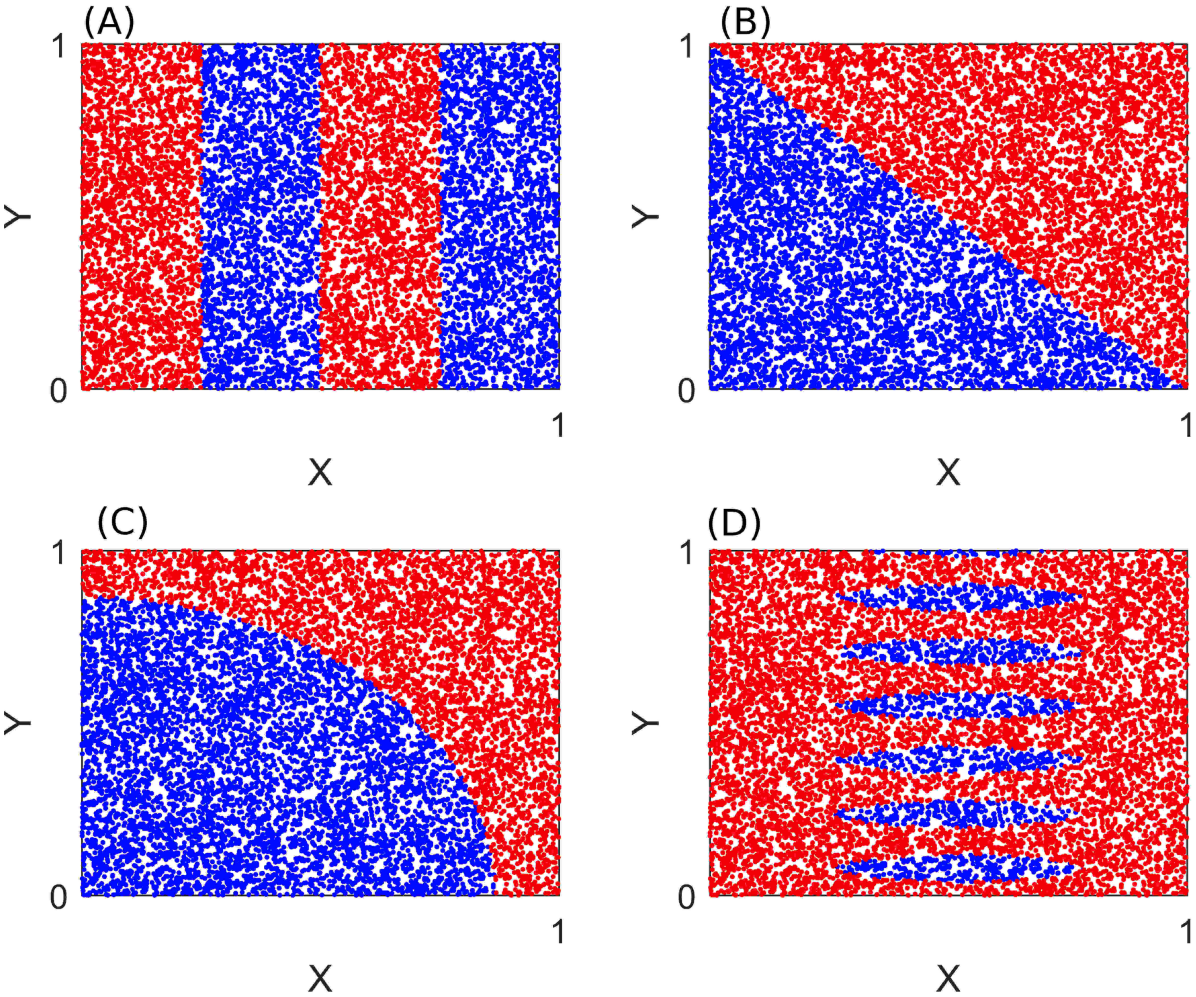
Example of the use of normalised variable importance (*NVI*), demonstrating that the *NVI* can identify the important parameters of different functions. Each subfigure shows evaluations of a different function (named *ex*_*a*_, *ex*_*b*_, *ex*_*c*_ and *ex*_*d*_, for details of these functions please refer to the main text) of three parameters: X, Y and Z. For each sampled parameter combination, the function outputs either 1 (red dots) or 0 (blue dots). Note that Z does not play a role in determining the output of any of these functions. (A) Simulation of *ex*_*a*_ over the parameter space, with resulting *NVIs*: *NVI*_*X*_ = 1, *NVI*_*Y*_ < 10^−3^ and *NVI*_*Z*_ < 10^−3^. (B) Simulation of *ex*_*b*_ over the parameter space, with resulting *NVIs*: *NVI*_*X*_ = 0.97, *NVI*_*Y*_ = 1 and *NVI*_*Z*_ < 10^−3^. (C) Simulation of *ex*_*c*_ over the parameter space, with resulting *NVIs*: *NVI*_*X*_ = 1, *NVI*_*Y*_ = 0.99 and *NVI*_*Z*_ < 10^−3^. (D) Simulation of *ex*_*d*_ over the parameter space, with resulting *NVIs*: *NVI*_*X*_ = 0.60, *NVI*_*Y*_ = 1 and *NVI*_*Z*_ = 0.02.

Fig 5 shows examples of *NVI* for some simple cases. The functions studied have three parameters *X*, *Y* and *Z*. The outputs are two classes, 0 or 1. The first function *ex*_*a*_ depends only on *X*, the functions *ex*_*b*_ and *ex*_*c*_ depend equally on *X* and *Y*. The last function, *ex*_*d*_, depends principally on *Y* and slightly on *X*.

$$\begin{array}{cc} ex_{a}\left(X,Y,Z\right)= & \{\begin{array}{ll} 0, & \text{if}\;0\leq X\leq 1/4\;|\;3/4\leq X\leq 1\\ 1 & \text{Otherwise} \end{array} \end{array}$$ (20)

$$\begin{array}{cc} ex_{b}\left(X,Y,Z\right)= & \left\{ \begin{array}{ll} 0, & \text{if}\;X+Y\leq 1\\ 1 & \text{Otherwise} \end{array}\right. \end{array}$$ (21)

$$\begin{array}{cc} ex_{c}\left(X,Y,Z\right)= & \left\{ \begin{array}{cc} 0, & \text{if}\;X^{2}+Y^{2}\leq 0.75\\ 1 & \text{Otherwise} \end{array}\right. \end{array}$$ (22)

$$\begin{array}{cc} ex_{d}\left(X,Y,Z\right)= & \left\{ \begin{array}{cc} 0, & \text{if}\;\cos^{2}\left(20X\right)+\sin^{2}\left(3Y\right)\leq 1\\ 1 & \text{Otherwise} \end{array}\right. \end{array}$$ (23)

The *NVI* is able to capture the importance of each parameter in each case. For example for the function *ex*_*a*_ the normalised variable importance of *X* is equal to one whilst the others are very close to 0. One can observe that the *NVI* of the parameter *Z* is not exactly equal to 0 even though the output of our function is independent from this parameter. The reason for this comes from the fact that in some trees, due to the sampling of our databases, the random forest can overfit a branch. This overfit branch will increase the variable importance of the independent parameters. Nevertheless this is a very marginal effect as one can observe in the examples of Fig 5. To check the convergence of *NVI* in our study we estimated the *NVI* of different parameters with a data base of 500,000 and 2,000,000 points. The maximum difference between the *NVI* of the two data bases was 0.05 which can be considered as negligible.

We implemented random forests using *r* [62] with the packages *RandomForest* [63]. The figures were built with *rpart* [64] and *rpart.plot* [65].

## Results

### Analyses of the data set

2,000,000 simulations were computed on the whole parameter space as described in section Wendling model, using the ranges in Table 2. Analysing these simulations, we found that the dynamics of the Wendling model can predominantly be categorised as steady state (62.3% of parameter space). The remaining simulations were classified by frequency and number of peaks (see Fig 3 for a description of dynamics). The dynamics ‘spike and wave’ or ‘poly-spike and wave’, which are characteristic of seizure dynamics, represent 5.8% of the parameter space. This number can be considered as the likelihood to find seizure dynamics when random parameters are used.

**Fig 6:**
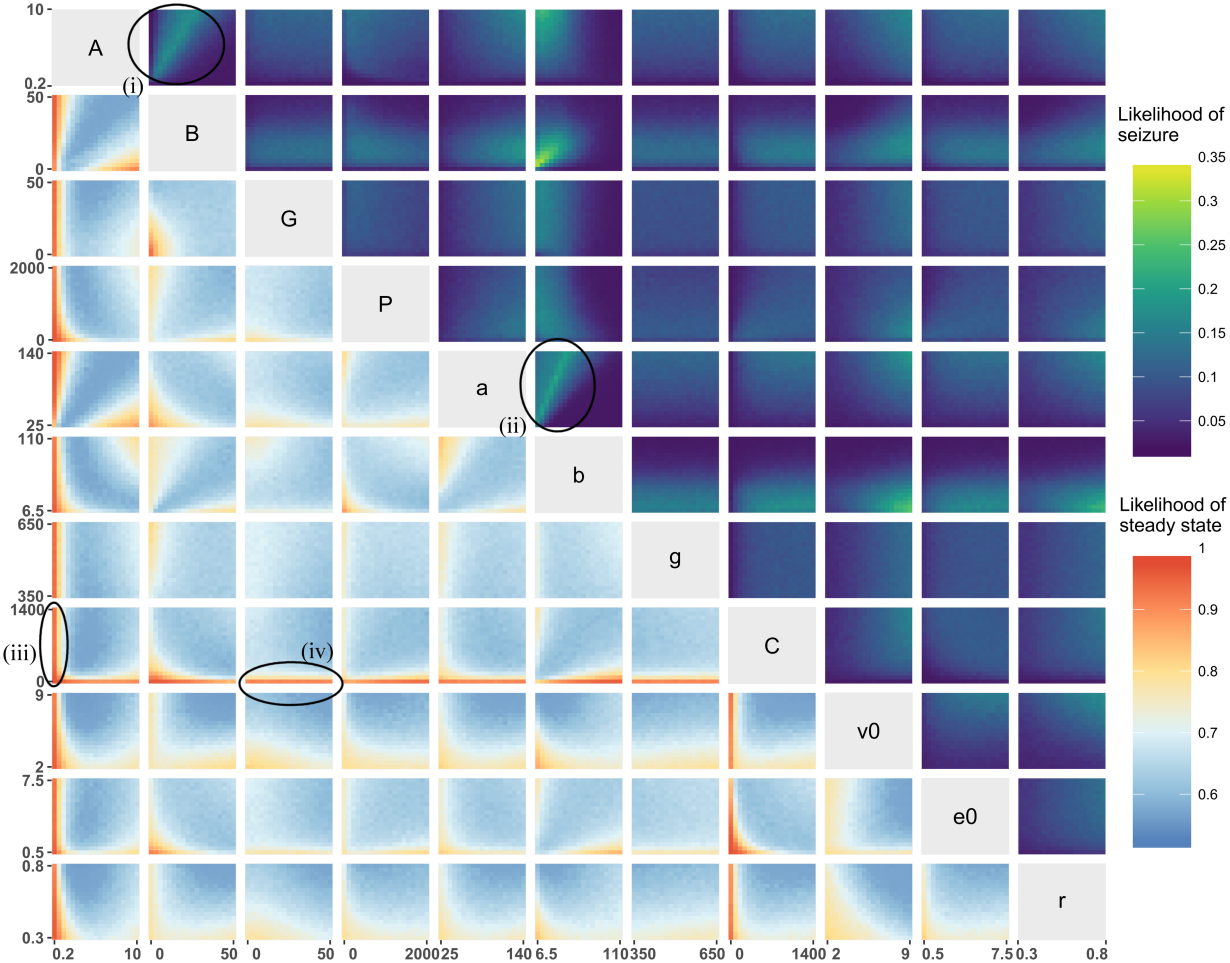
Bivariate joint distribution of the likelihood of steady state (lower triangle) or seizure dynamics (upper triangle). Each subfigure is a projection of the parameter space over two parameters, and the colour indicates the likelihood of finding a particular type of dynamics (seizure or steady state) as per the colourbar. For example, the subfigure in the second column on the first row (encircled and labelled (i)) maps the likelihood of finding seizure dynamics over different values of *B*(x-axis) and *A*(y-axis), given variations in all other parameters. In the upper triangle, yellow indicates high likelihood of observing seizure dynamics, whereas blue indicates low likelihood of observing seizure dynamics. In the lower triangle, red indicates high likelihood of observing steady state dynamics, whereas blue indicates low likelihood of observing steady state dynamics. Each subfigure was computed using eq 12 with 20 × 20 bins over the parameter ranges provided in Table 2. Upper triangle: Specific combinations of parameters can lead to manifolds with a high likelihood of seizure dynamics (see for example the linear relationships between *A* and *B* in the encircled subfigure (i) and *a* and *b* in the encircled subfigure (ii)). Lower triangle: one can observe that small values of the parameters *A* or *C* guarantee a steady state (see for example the encircled subfigures (iii) and (iv)).

Fig 6 provides a 2-dimensional representation of the distribution of steady state and seizure dynamics throughout the whole parameter space. It allows us to detect the combination of parameters that are particularly prone to producing seizure dynamics or steady states in the model. It can be seen that the parameter subspace in which seizure dynamics can be found is large and is not concentrated in small sub-regions. The top right of Fig 6 demonstrates that seizure dynamics can be observed across most parameter values; there are few combinations of two parameters for which, regardless of other parameter values, seizure dynamics cannot exist. Examples are the inverse mean time in the excitatory and slow inhibitory loop (*a* and *b*), which give rise to dark blue regions in Fig 6 (low likelihood of seizure dynamics). Specific combinations of parameters *A*, *B*, *C*, *e*_0_ or *r* can also preclude seizure dynamics. In contrast, the subfigures for parameters of the fast inhibitory loop (*G* and *g*) appear quite homogeneous, and therefore do not change the likelihood of seizure dynamics.

However, varying the value of some parameters does reduce the likelihood of observing seizure dynamics: reducing parameters of the excitatory loop (*A* and *a*); the connectivity coefficient (*C*); the maximum firing (*e*_0_); and the inflexion point (*v*_0_) and the slope (*r*) of the sigmoidal nonlinearity. On the other hand, increasing the inverse mean time in the slow inhibitory loop (*b*) also reduces the probability of observing seizure dynamics. Intermediate values of the input (*P*) and the average slow inhibitory gain (*B*) increase the chance of observing seizure dynamics. Particular combinations of pairs of parameters such as the average synaptic gains (*A* and *B*) or the inverse time scales (*a* and *b*) can significantly alter the chance of observing seizure dynamics. For example, there is a linear combination of *a* and *b* for which the proportion of dynamics in the seizure class is greater than 30%. The lower triangle of Fig 6 indicates that steady state dynamics can be observed in a very large proportion of the parameter space. It can be seen that small values of *A* or *C* force the system to be at steady state.

**Fig 7:**
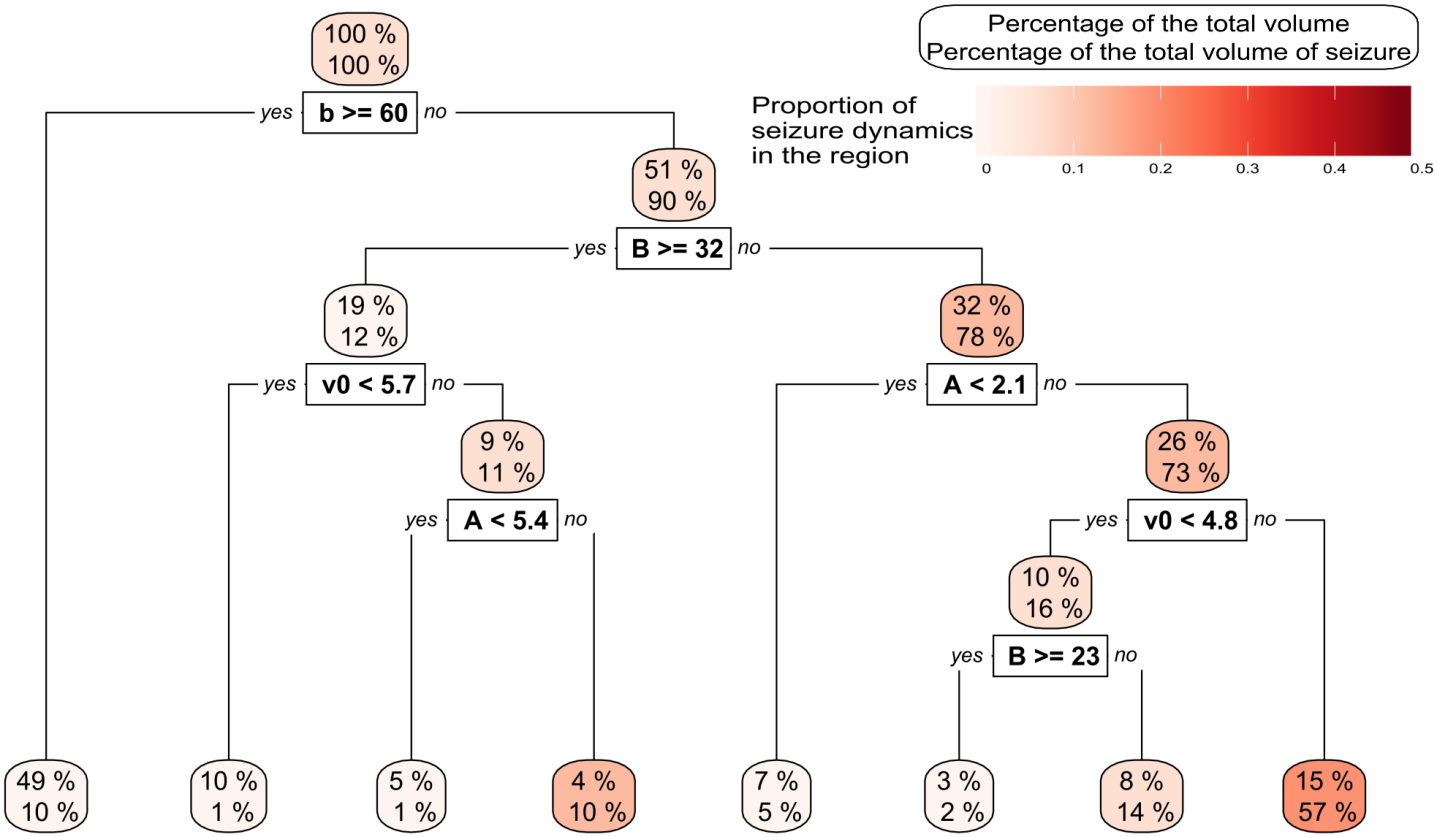
A tree representing how parameter space is split dependent on the presence or absence of seizure dynamics. The root region is at the top of the figure and represents 100% of the parameter space while the leaves are at the bottom. The upper label of each regions indicates the size of the parameter space represented in this region. The lower label indicates the percentage of all parameter combinations that result in seizure dynamics. The colour indicates the density of seizure dynamics in the given region. The parameters *A*, *B*, *a* and *b* are the most important parameters because they efficiently split the parameter space into subspaces with high or low likelihood of seizure dynamics. Some parameters such as *P* and *C* do not appear in this small tree, however they can appear in a more complex tree (see supplementary material, S1 Fig). Values are given to two digits precision.

Explorations such as undertaken in Fig 6 are informative and give a good preliminary indication of the role that each parameter plays in constraining the model dynamics. Nevertheless, in more than two dimensions, visualisation becomes difficult. For example, extending Fig 6 to 3 dimensions would require 1,000 2D plots. Therefore, we used tree statistics (see section Building a tree) to efficiently summarise how a change in a parameter can impact the dynamics of the model. Fig 7 presents one such tree that describes the segmentation of parameter space according to the density of seizure dynamics. Recall, that for each branch the tree algorithm scans through the sub-parameter space to identify the optimal separation between the maximum and minimum likelihood of observing the feature of interest (seizure dynamics in this case).

Fig 7 is a relatively small tree used to illustrate the method. At the root of this tree, the first parameter used to partition parameter space is the inverse time scale of the slow inhibitory loop, *b*. *b* ≥ 60 reduces the probability of observing seizure dynamics and produces a region that represents 49% of the parameter space. This region, which represents nearly half of the parameter space, contains only 10% of all parameter combinations that lead to seizure dynamics. Taking *b* < 60 again yields approximately half of the total parameter space (51%), but this region contains 90% of all parameter combinations that lead to seizure dynamics. Since this region is large, and the probability of observing seizures in the whole space is low (5.8%), the *density* of seizures in this region is low at 10%. The next branch cuts through the average slow inhibitory gain at *B* = 32. Above this value, 19% of the parameter space remains and this contains 12% of all parameter combinations that yield seizure dynamics. The remaining 32% of parameter space accounts for 78% of seizure dynamics. Choosing *A* ≥ 2.1 further increases the density of seizure dynamics to 17%, incorporating 73% of all parameter sets that lead to seizure dynamics. Further adding the criterion that *v*_0_ ≥ 4.8 leads to a region with highest density of seizure dynamics (bottom right region in Fig 7). This region represents 15% of the total parameter space and the proportion of seizure dynamics in this region is 22%; thus it accounts for 57% of all parameter combinations that result in seizure dynamics.

However it is possible to create larger trees with more regions giving a finer resolution. There is of course a trade-off as larger trees segment the parameter space into more (smaller) hypercubes, making them more cumbersome to analyse (see supplementary materials S1 and S2 Figs for more examples). The main conclusion to be drawn from the large tree presented in the supplementary material is that the dependency of dynamics on parameter space is complex: transitions between dynamics can vary between regions. For example, an increase of *B* or *P* can either increase or decrease the likelihood of seizure dynamics. However, other parameters exhibit robust transitions; a split at *r* around 0.52 appears consistently, and *e*_0_ and *v*_0_ tend to slightly increase the seizure likelihood when their values increase.

Fig 6 seems to show different results from [11]. To recall; in [11], the presence of seizure dynamics would appear only for B superior to 20 and *A* superior to 5 (other parameters at standard value as in Table 2). In contrast, in our Fig 6 the likelihood of seizure when *B* is superior to 20 is low and higher for small values of *B*. These results illustrate that although a projection of the parameter space in 2-dimensions is helpful to gain a quick understanding of the parameter space, it does not capture all of its aspects. In Fig 7, with the help of the tree algorithm, the manifold is well approximated. Indeed one can see that even for large *B* (*B* > 32) seizure dynamic can appear with the standard values of parameters [11] (fourth leaf from the left). Furthermore the tree shows that this change appears around *A* = 5.4. There are other, larger manifolds in Fig 7 at small values of *B*. These manifold are the ones which influence Fig 6 the most and “mask” the results of [11].

### Determining the relative importance of parameters for observing features of interest

To generalise the example of Fig 7, we computed the variable importance of model parameters over a random forest of 100 trees. Clearly, the importance of a parameter depends on the characteristics we are interested in. Results regarding the presence of steady states, oscillations with different amplitudes and frequencies, as well as seizure dynamics are provided in Table 3. We find that *A*, *B*, *C* and *v*_0_ are important parameters for transitioning between steady state dynamics and the different types of oscillations. Interestingly, the amplitude of oscillations was less dependent on *A* and instead strongly dependent on *C* and *e*_0_. This might seem surprising given the importance of *A* in observing oscillations in the first place. This contrast demonstrates how the relative importance of a parameter is strongly dependent on the observed feature of interest (e.g. frequency vs the amplitude). The input from other regions of the cortex (*P*) can affect the emergence of oscillations but has a marginal role in tuning the amplitude and the frequency of these oscillations. The connectivity constant (*C*) is important for governing the amplitude but not the frequency of an oscillation. In fact, few parameters (*A*, *B*, *a*, *b* and *G*) are important for determining the frequency of oscillations.

**Table 3:** The importance of parameters as determined by normalised variable importance (*NVI*) averaged over a random forest of 100 trees. Four characteristics of interest are considered: the switch between steady state and non-steady state, the amplitude of cycles, the frequency of cycles and the switch between any activity (mainly steady state) and seizure dynamics. A value of 1 signifies the parameter with greatest importance for observing the feature of interest (e.g. *A* is most important for observing transitions from steady-state). A value of 0 implies a parameter has no control over observing a feature of interest.

	A	B	G	P	a	b	g	C	v_0_	e_0_	r
steady-state to cycle	1	0.40	0.11	0.15	0.27	0.32	0	0.59	0.48	0.16	0.26
amplitude of oscillation	0.13	0.46	0	0.02	0.13	1	0	0.96	0.11	0.83	0.04
frequency of oscillation	0.14	1	0.09	0.01	0.11	0.86	0.01	0.01	0.01	0.01	0
transition to seizure dynamics	0.25	0.59	0.03	0.09	0.30	1	0	0.22	0.28	0.07	0.15

All parameters except *g* were found to play a role in the generation of transitions between dynamics, but with varying importance. The frequency of an oscillation was found to be predominantly dependant on the inhibitory slow loop parameters (*B* and *b*). These parameters were also found to be crucial for producing seizure dynamics. This observation confirms the finding in Fig 7 that when these parameters split the space they reduce impurity. Overall the excitatory pair of pyramidal and excitatory interneurons and the slow inhibitory loop are important to create oscillations in the Wendling model. The output of the Wendling model is sensitive to a change of any of these parameters as indicated by the *NVI* measurements (Table 3).

### Extension to parameter ratios

**Fig 8:**
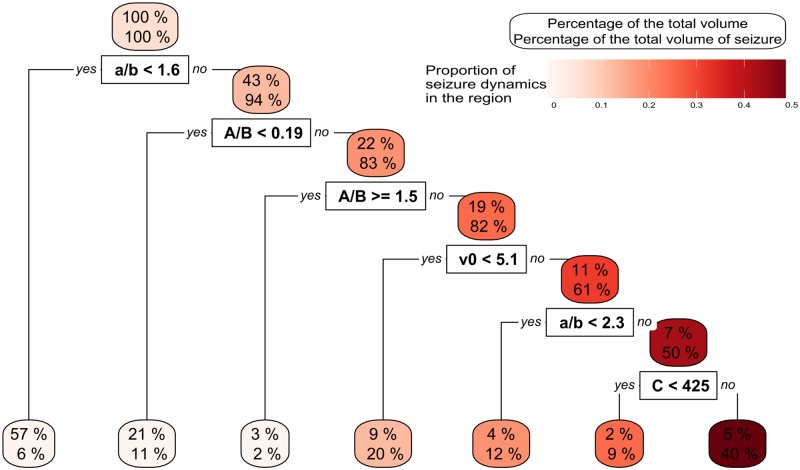
A tree representing how the extended parameter space (incorporating two additional ‘ratio parameters’ *r*_*a*/*b*_ and *r*_*A*/*B*_) is split dependent on the presence or absence of seizure dynamics. The root region is at the top of the figure and represents the total parameter space, while the leaves are at the bottom. The upper label of each region indicates the size of the parameter space represented in this region. The lower label indicates the percentage of all parameter combinations that result in seizure dynamics. One can see that the ratios have an important role in splitting the parameter space. Values are given to two digits precision.

Fig 6 demonstrated a potentially important relationship between the parameters *A* and *B* and the parameters *a* and *b*. We further investigated this by incorporating two artificial parameters *r*_*A*/*B*_ and *r*_*a*/*b*_ which are respectively the ratio of *A* over *B* and the ratio *a* over *b*. Fig 8 shows that smaller values of *r*_*a*/*b*_ lead to a lower likelihood of observing seizure dynamics. A ratio less than 1.6 gives a likelihood of observing seizure dynamics of 0.56% in a very large sub-region that contains 57% of the parameter space. At the opposite extreme, the region on the right of the figure contains 40% of all seizure dynamics in only 5% of the whole parameter space. In this region the proportion of seizures is nearly 50%. It is interesting to note that low values of *r*_*a*/*b*_ reduce the likelihood of seizure dynamics, whereas for *r*_*A*/*B*_, small values (<0.19) or large values (>1.5) reduce the likelihood of seizure dynamics. A more highly resolved version of this tree can be found in supplementary materials S2 Fig. We recomputed the *NVI*, incorporating these two new parameters over a random forest of 100 trees. The results are in Table 4. It is clear that for steady state transitions or frequency of oscillations *r*_*A*/*B*_ is the most important, whereas *r*_*a*/*b*_ is most important for transitions to seizure dynamics. Aside from the amplitude of oscillations, the normalised variable importance of the ratios *r*_*A*/*B*_ and *r*_*a*/*b*_ are larger than for the parameters taken individually.

**Table 4:** The importance of parameters as determined by normalised variable importance (*NVI*) averaged over a random forest of 100 trees. The ratios *r*_*A*/*B*_ and *r*_*a*/*b*_ have been added as additional parameters. Four characteristics of interest are considered: the switch between steady state and non-steady state, the amplitude of cycles, the frequency of cycles and the switch between any activity (mainly steady state) and seizure dynamics. A value of 1 signifies the parameter with greatest importance for observing the feature of interest. A value of 0 implies a parameter has no control over observing a feature of interest.

	A	B	G	P	a	b	g	C	v_0_	e_0_	r	r_*A*/*B*_	r_*a*/*b*_
steady-state to cycle dynamics	0.65	0.24	0.13	0.17	0.16	0.19	0	0.62	0.49	0.17	0.25	1	0.57
amplitude of oscillation	0.10	0.59	0.01	0	0.1	0.89	0	1	0.07	0.89	0.02	0.14	0.15
frequency of oscillation	0.04	0.51	0.04	0	0.11	0.71	0.01	0.01	0	0	0	1	0.41
transition to seizure dynamics	0.11	0.33	0.04	0.12	0.06	0.3	0	0.14	0.31	0.08	0.17	0.8	1

## Discussion

In this study, we introduced a new approach to explore the parameter space of high dimensional NMMs. In contrast to classical studies that considered parameters individually, or in pairs, we used a random forest approach in order to study the entire parameter space simultaneously.

Our approach relies on the creation of a database of dynamic features derived from forward simulations. Other statistical approaches could be used to study the database, but they all suffer from particular deficiencies. For example, artificial neural network models have a vast number of hyperparameters that cannot be interpreted [66]. Support vector machines [67] result in boundaries between regions of parameter space that are not split according to single parameters, and therefore one has to integrate over all parameters to understand the importance of each. Kernel methods such as Gaussian processes [68] rest upon the assumption of “smoothness” of data, i.e., proximal parameter sets are assumed to yield similar simulations, which is clearly not the case close to bifurcations. Another approach combines trees and Gaussian process [69, 70], but that approach requires prior assumptions on parameters, limiting its use when this information is not to hand. In contrast, the approach we employed provides an efficient way to study the influence of model parameters on their dynamics: trees are computationally fast, make no *a priori* assumptions on either the type of model or parameter values, and can handle data that are represented on different measurement scales [71]. We thereby demonstrated that random forests are a useful tool to study the dynamics of NMMs.

The implementation of the random forest approach [56], overcomes the issue that each implementation of CART produces a single tree that is locally optimal. A drawback is that the random forest approach introduces some loss of interpretability, but the final solution is more representative of the global optimum. This is particularly important for the *NVI* which measures the relative contribution of parameters to an observed dynamic feature of interest of the model (e.g. a steady-state, oscillation or spike-wave). By this we mean that effectively, the *NVI* indicates which parameters are critical for segmenting the total parameter space into regions in which a feature of interest is more or less likely to be observed. Further, the *NVI* provides a principled approach for determining whether or not parameters can be fixed, hence reducing the number of parameters to be calibrated from observable data. A consistently low *NVI* across all features of interest means that the considered parameter plays little role in any dynamic change and can therefore be fixed to an arbitrary value within a given physiological range.

For example, in our study of the Wendling model, *g* has little effect on determining transitions from steady-state to oscillations, or in determining the amplitude and frequency of those oscillations. It can therefore be fixed, meaning that the parameter space explored in subsequent calibration is smaller. On the other hand, some parameters have a high *NVI* for specific features of interest, and are therefore important for observing that specific feature without playing an important role in altering other aspects of the dynamics. For example, *e*_0_ is critical in determining the amplitude of oscillations, but plays a marginal role in the appearance of other features. Therefore if amplitude is not a particular feature of interest, *e*_0_ could be fixed. When considering networks of dynamical systems, the number of parameters can rapidly become very large, so *NVI* is an important tool for managing this increase in complexity. For example, one could use the framework presented herein to examine whether there are certain network structures in which certain edges can be given fixed weights, thereby reducing the dimension of an optimisation or calibration problem.

The notion of importance is defined using *NVI* due to its robustness and ability to measure the influence of parameters on dynamics [72]. However, the notion behind importance is somewhat nebulous, and it is difficult to directly attribute a small difference in *NVI* to the relative importance of a specific parameter. The pragmatic approach we have adopted, is to consider parameters with values of *NVI* > 0.1 as playing a role in governing the feature of interest. In contrast, parameters with a *NVI* close to 0 can be disregarded. In the present study we defined importance specifically in the context of changes in parameters causing changes in asymptotic dynamics. This is relevant for the case in which bifurcations give rise to epileptiform activity. However, there are other possible model scenarios in which changes in dynamics could occur, such as for example, intermittency, bistability and excitability [73]. In these cases, we would seek to characterise importance with respect to changes in unstable invariant sets of the system, for example boundaries of basins of attraction. Furthermore, importance as we have defined it in the context of the NMM does not imply that a parameter is crucial for changes in dynamics at the individual level. For example, it might be necessary to model some seizures using transitions between dynamics that occur only in small regions of parameter space. It is important to highlight that in the random forest approach, other definitions of importance exist, such as the permutation importance or the conditional permutation importance [61]. However, these approaches suffer from lack of robustness [74], hence our focus on *NVI*.

Our analyses of the full parameter space of the Wendling model show that parameters of the slow inhibitory loop (*b* and *B*) play the most important role (in term of *NVI*) in the emergence of seizures. The time scale of the slow inhibitory loop (*b*) is the most important parameter; a small change in its value can transform steady state dynamics into seizure dynamics robustly, i.e. for the majority of combinations of other parameters in the model. We found the excitatory loop, governed by *a* and *A*, together with the offset of the sigmoid function (*v*_0_) to be the second most important components of the system for the emergence of seizure dynamics. These are followed by the other parameters of the sigmoid function (*v*_0_ and *r*) and the parameter that scales connectivity between the different populations of neurons (*C*). Interestingly, changes in the fast inhibitory loop (parameters *g* and *G*) do not play an important role in the generation of seizure dynamics. We note that a low value of *NVI* in the context of our study does not mean that a parameter is irrelevant to the emergence of other brain dynamics not captured by the choice of features. Furthermore, parameters with low *NVI* may play a role in determining transitions between dynamics in specific subsets of parameter space; *NVI* is purely a global measure. The parameters governing the magnitude of input from other areas (*P*) or the scaling of intrinsic connectivity (*C*), for example were shown herein to have little (global) effect on the emergence of seizure dynamics, but in *a priori* constrained sub-regions have been shown capable of governing transitions in NMMs [7, 8]. Table 4 shows a comparison of parameter importance when different features are considered. Parameters of the slow inhibitory loop, *b* and *B*, as well as the ratio of time scales *r*_*a*/*b*_, showed relatively high importance across all features. It is therefore possible that these parameters are important for transitions between dynamics in general. Verifying this will require exploration of additional features in model dynamics.

We found that the ratio of parameters of the excitatory and inhibitory loops play an important role in the generation of all the features we considered, with the exception of amplitude of oscillations. The ratio of time scales (*r*_*a*/*b*_) is the most important factor governing emergence of seizures, whereas the ratio of gains (*r*_*A*/*B*_) is most important for the onset of cycles and the frequency of these cycles. Reducing *r*_*a*/*b*_ robustly reduces the likelihood of seizures regardless of other parameter values (see e.g. Fig 8 and supplementary materials, S2 Fig). *r*_*A*/*B*_ on the other hand, presents an intermediate range of values that have highest likelihood of seizure dynamics.

Our finding of the importance of *r*_*A*/*B*_ for the emergence of seizure dynamics is in line with previous experimental observations. For example, [75] found that the ratio of Glutamine to GABA Levels is larger in people with idiopathic generalized epilepsies compared to healthy controls. This also aligns with the action of some antiepileptic drugs, for example those acting via modulation of neurotransmitters such as GABA [76], the potentiation of which would be reflected in our model by an increase in *B*, and hence a decrease in *r*_*A*/*B*_. Furthermore, Our finding of the importance of *r*_*A*/*B*_ for the emergence of seizure dynamics confirms previous modelling results [30]

Interestingly, since the highest likelihood of emergent seizure dynamics was found to be for intermediate values of *r*_*A*/*B*_, this would suggest that, depending on the choice of other parameters, *decreasing* the ratio of excitation to inhibition could also produce a route into seizure dynamics, in line with evidence of the possibility of heightened inhibition at seizure onset [77]. Our finding that the slow inhibitory and excitatory synaptic gains have more influence than the fast inhibitory loop is in line with previous modelling results [33, 34], as are our findings that the parameter *r* and the ratio *r*_*a*/*b*_ are important for dynamics of the NMM [35, 78, 79].

Few experimental studies have investigated the role that different time constants might play. However, it has been shown that chloride ion homeostasis is perturbed in patients with mesial temporal lobe epilepsy [80], and intracellular chloride ion concentrations have been shown to play a role in the time constants of postsynaptic potentials [81]. This therefore presents a possible biophysical interpretation for the importance of *r*_*a*/*b*_. Interestingly, a recent study utilising dynamic causal modelling applied to a zebrafish model of seizures also demonstrated the potential importance of excitatory and inhibitory synaptic time constants [82].

In our study, we obtained these results using a method in which the influence of all parameters was analysed simultaneously and a complete characterisation of the relative importance of all parameters was possible. In fact, this analysis revealed new combinations of parameters that can potentially govern the emergence of seizure dynamics in the Wendling model, for example *v*_0_. In addition, given our finding that the ratio *r*_*a*/*b*_ is most important for seizure generation it would be interesting to explore the known effect of drugs that could target the inverse mean time ratio *r*_*a*/*b*_.

[11] presented detailed, two-dimensional analyses of the effects that changing system parameters have on emergent dynamics. One of the findings of [11] was that seizure dynamics predominantly occur when *B* > 20. However, our results (Fig 6) show that the likelihood of seizures when *B* > 20 appears rather low (but not zero) and is in fact higher for small values of *B*. These results indicate that although a projection of the parameter space in 2-dimensions is helpful to gain a quick understanding of the system, it does not capture the global picture. In our Fig 7, with the help of the tree algorithm, we did indeed find that for large *B* (>32) seizure dynamics occur for the range of parameters used by [11] (fourth leaf from the left in Fig 7). Furthermore the tree shows that this change appears around *A* = 5.4. However, our analysis in Fig 7 demonstrates that there are other regions of parameter space, for lower values of *b* that contain seizure dynamics.

The approach presented herein relies on the construction of a statistical model of dynamics based on simulations. This means that we cannot uncover the dynamic mechanisms that govern the emergence of the features studied, for example the presence of unstable invariant sets or changes in stability. However, our approach could be combined with traditional methods such as numerical continuation [83]; we would first constrain parameter space by using *NVI* to identify the most important parameters, together with transition boundaries and then perform more detailed analyses therein.

Studies including [73] and [84] describe four alternative mathematical mechanisms underlying the emergence of seizures: bifurcation (a parameter is slowly varied so that the system crosses a bifurcation point), bistability (backround and seizure attractors co-exist, with perturbations allowing transitions between the two), transient excitability (the seizure dynamics occurs due to a complex trajectory elicited by a perturbation) and intermittency (background and seizure dynamics are part of the same attractor). In this study we have focussed on a detailed explanation of the bifurcation mechanism (e.g. how small changes in system parameters can lead to abrupt changes in emergent dynamics). Specifically, we find for the chosen Wendling model that under the bifurcation assumption changes in the slow inhibitory loop or the excitatory loop are most likely to underpin the emergence of seizures. It is important to highlight that this finding is specific to the chosen model and further, that it does not exclude the other three possibilities. To explore the possibility of transient excitability and bistability, we would need to extend our statistical model to include system variables (e.g. initial conditions) and properties of perturbations as parameters. We investigated the impact of initial conditions by considering them as parameters and found their *NVI* to be close to zero, indicating that regions of bistability are small in the context of global changes in parameters.

Another possible extension to the results presented herein would be to consider different dynamic models or different characteristic features of their dynamics. For example [85] or [86] focused their attention on the power spectrum of the model in comparison with clinically recorded data. Future work could focus on power spectra as a feature of interest, enabling an appropriate characterisation of the importance of parameters for generating alpha activity in NMMs.

In summary, we presented a framework for the global characterisation of the dynamics of NMMs. Our methods have the potential to advance patient-specific model representations, for example by first determining the relative importance of parameters, and then reducing the parameter space to a subset in which model calibration from data becomes tractable. Such an approach will become increasingly important as the emphasis on networked dynamical systems of the brain increases. Here the number of model parameters grows rapidly, beyond the point for which established approaches such as Kalman filtering [15] or genetic algorithms [32], that work directly with the dynamical system of interest, can be effective.

## Supporting information

S1 Code Code working with matlab R2017a and R 3.40. Code to generate the different results of the article.
(ZIP)

S1 Fig Large tree. The parameter space is split dependent on the presence or absence of seizure dynamics. The figure represents a tree with all the parameters and with a minimum size of leaf of 1000 simulations.
(PDF)

S2 Fig Large tree with modified parameter space. The parameter space is split dependent on the presence or absence of seizure dynamics. The figure represents a tree with all the parameters and the ratio of the parameter *A* over *B* and *a* over *b*. The minimum size of leaf is 1000 simulations.
(PDF)
