# Supplementary figures and images for: Classifying dynamic transitions in high dimensional neural mass models: A random forest approach
Source: PLoS Comput Biol. 2018 Mar 2;14(3):e1006009. doi: 10.1371/journal.pcbi.1006009 (PMC5851637; doi:10.1371/journal.pcbi.1006009)

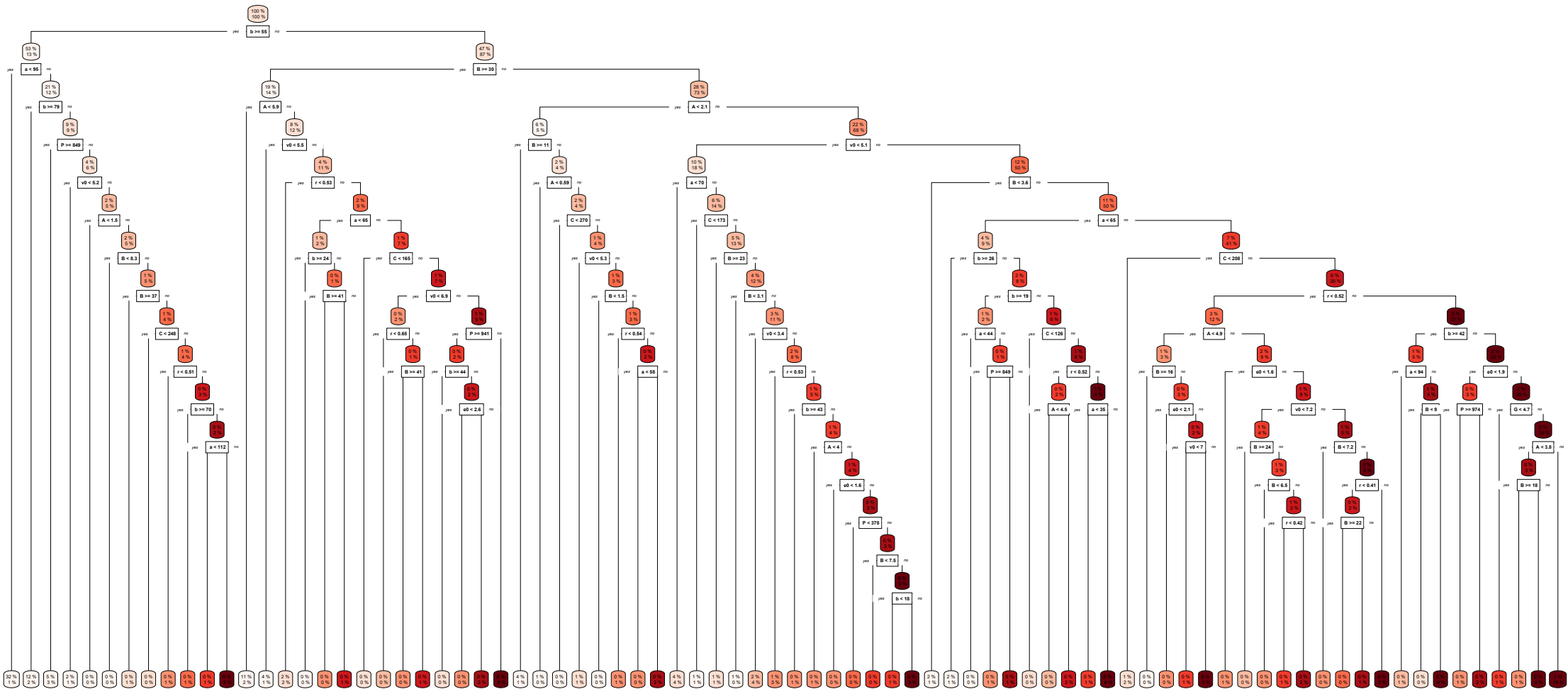

Supplement: S1 Fig — The parameter space is split dependent on the presence or absence of seizure dynamics. The figure represents a tree with all the parameters and with a minimum size of leaf of 1000 simulations. (PDF) [file pcbi.1006009.s002.pdf]

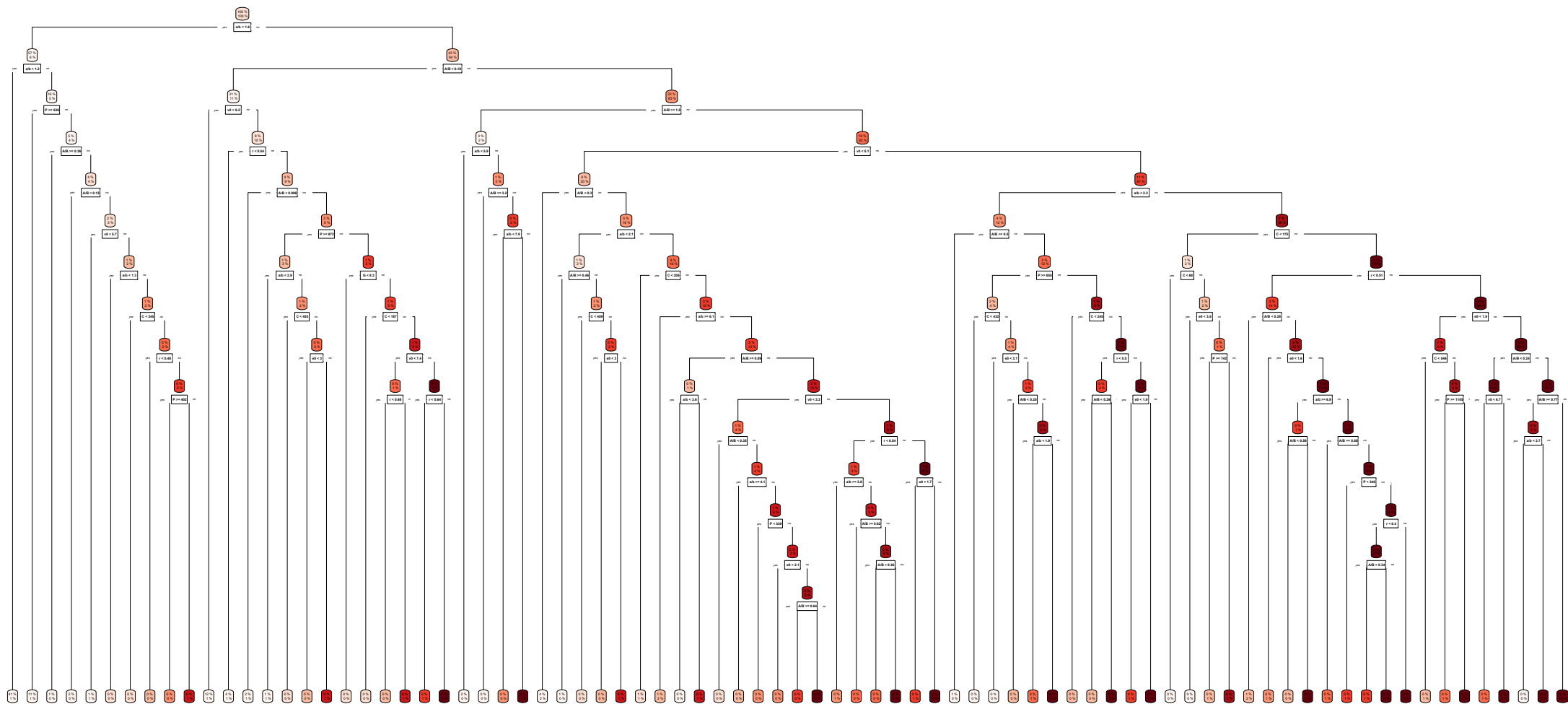

Supplement: S2 Fig — The parameter space is split dependent on the presence or absence of seizure dynamics. The figure represents a tree with all the parameters and the ratio of the parameter A over B and a over b. The minimum size of leaf is 1000 simulations. (PDF) [file pcbi.1006009.s003.pdf]
